# The Architectural Dynamics of the Bacterial Flagellar Motor Switch

**DOI:** 10.3390/biom10060833

**Published:** 2020-05-29

**Authors:** Shahid Khan

**Affiliations:** Molecular Biology Consortium, Lawrence Berkeley National Laboratory, Berkeley, CA 94720, USA; khan@mbc-als.org

**Keywords:** rotary molecular motor, protein allostery, chemotactic signaling

## Abstract

The rotary bacterial flagellar motor is remarkable in biochemistry for its highly synchronized operation and amplification during switching of rotation sense. The motor is part of the flagellar basal body, a complex multi-protein assembly. Sensory and energy transduction depends on a core of six proteins that are adapted in different species to adjust torque and produce diverse switches. Motor response to chemotactic and environmental stimuli is driven by interactions of the core with small signal proteins. The initial protein interactions are propagated across a multi-subunit cytoplasmic ring to switch torque. Torque reversal triggers structural transitions in the flagellar filament to change motile behavior. Subtle variations in the core components invert or block switch operation. The mechanics of the flagellar switch have been studied with multiple approaches, from protein dynamics to single molecule and cell biophysics. The architecture, driven by recent advances in electron cryo-microscopy, is available for several species. Computational methods have correlated structure with genetic and biochemical databases. The design principles underlying the basis of switch ultra-sensitivity and its dependence on motor torque remain elusive, but tantalizing clues have emerged. This review aims to consolidate recent knowledge into a unified platform that can inspire new research strategies.

## 1. The Problem Framed—Historical Background (1973–2003)

**Figure 1:**
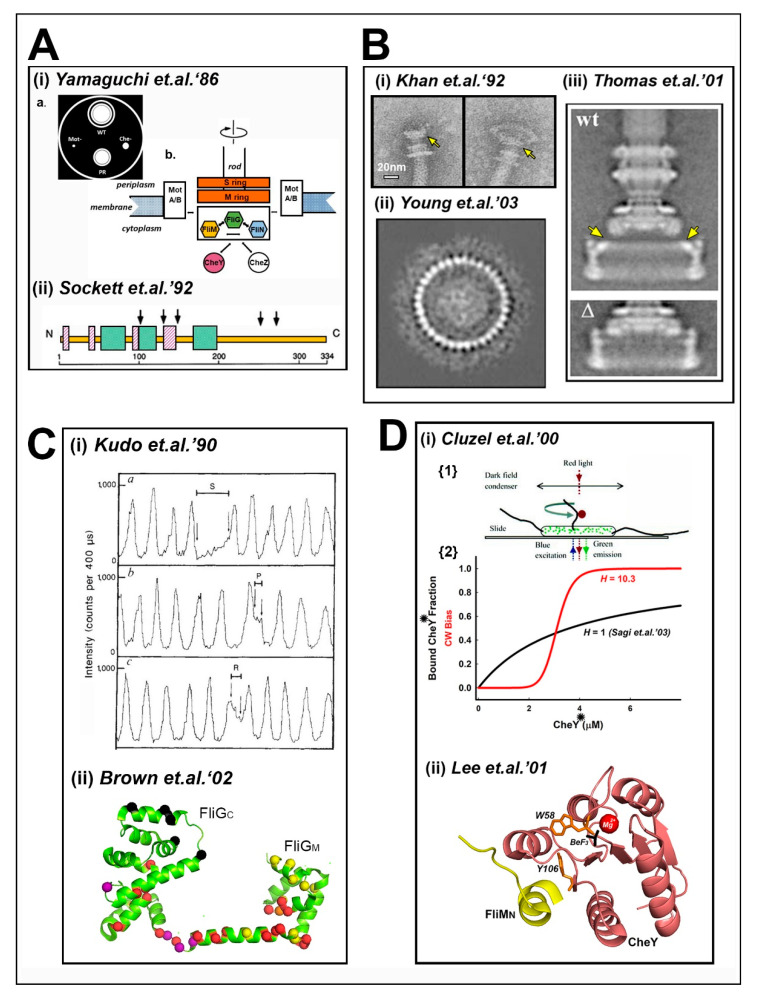
The bacterial flagellar motor (BFM) switch—landmarks. (**A**) Conceptualization: (**i**) The switch complex was proposed based on phenotypic characterization of *mot*, *che* and *fla* alleles and their suppressor mutations in swarm plate assays. Its interactions with chemotaxis components and Mot proteins were also identified. {**a**} Schematic of a swarm plate—the native (WT) strain forms a swarm with chemotactic rings. Strains carrying *mot* mutations (Mot-) do not swarm while those with *che* mutations (Che-) have reduced swarms. Suppressor mutations yield pseudo-revertant strain (PR) with partially restored swarming. {**b**} Color codes are followed in subsequent Figures for the switch complex components (FliG (green), FliM (gold), FliN (cyan)), the CheY protein (salmon) and the MS-ring scaffold (orange) (adapted from [6]). (**ii**) Gene sequencing identified the mutations. The *fliM* gene (N–C terminal residue numbers) predominantly contained the *che* lesions, clustered into distinct CW (green) and CCW (magenta) regions. Arrows mark *mot* lesions (adapted from [8]). (**B**) Structural identification: (**i**) An extended cytoplasmic structure contiguous with the basal body MS-ring (yellow arrow) was isolated using gentler protocols and subsequently established as the switch complex by immuno-EM and biochemistry (from [14]). (**ii**) Assembly of switch complex by overproduction of plasmid-encoded components allowed biochemical characterization culminating in the determination of the C ring subunit stoichiometry (n = 33–34) (from [23]). (**iii**) Single-particle analysis resolved FliG domain substructure (yellow arrows) from differences in central sections from wild-type (WT) and ∆FliFFliG (∆) 3D basal-body reconstructions (from [22] with permission). (**C**) Motor function and mechanism: (**i**) Temporally resolved measurement of filament rotation, as a sinusoidal variation of laser dark-field spot intensity, characterized aberrant phenotypes in switch complex mutant strains. Panels (top to bottom) show slow rotation (S), pausing (P) and reversal (R) episodes (reproduced from [32] with permission). (**ii**) The first atomic structure of a switch component (FliGc [33]) followed by the FliG_MC_ structure localized much of the mutant library then available ((mot lesions (black); CW lesions (red); CCW lesions (yellow); CW or CCW, depending on the residue substitution, orange; and *motB* suppressors (purple)) to generate chemically explicit ideas for motor reversal (PDB: 1lkv (modified from [34])). (**D**) Switch chemotactic signal transduction: (**i**) {1}—Determination of switch “ultra-sensitivity” (Hill coefficient, *H* = 10.3) by simultaneous measurement of the CW bias of beads on flagellar stubs (red) and concentration of a fluorescent GFP-CheY fusion (green) locked in the active state (*) in engineered strains (reproduced from [45] with permission). {2}—Plots show non-cooperative binding of acetate-activated CheY to overproduced C rings [46] compared to the in-vivo change in CW bias. (**ii**) The atomic structure of beryllium-fluoride (BeF3 (black))-activated CheY (salmon) bound to the FliM N-terminal peptide (yellow) initiated structure guided mutagenesis to explain the switch ultra-sensitivity. Aromatic residue (W58, Y106 (orange)) motions were early diagnostics for activation. Magnesium ion (red) (PDB: 1f4v (modified from [41])).

Bacterial motility has been a long-standing example of motion on a microscopic scale [1]. The modern era began with the realization that bacterial flagella rotate, as opposed to eukaryotic flagella that beat [2]. The fundamental issues that drive current research on the bacterial flagellar switch were framed in the first thirty years (1973–2003). The first stage, the “classical period”, established that the energy source for motility was the chemiosmotic ion potential rather than ATP. Tethered cell assays demonstrated cell rotation driven by a single flagellum immobilized on glass coverslips. These assays showed that eubacterial motors rotate both counterclockwise (CCW) and clockwise (CW) and switch rotation sense without a detectible change in rotation speed. The CW and CCW rotation intervals were Poisson distributed. Chemo-effectors changed motor rotation bias with sub-second excitation followed by adaptation over seconds back to the pre-stimulus level. Motor rotation bias (CW/(CW + CCW)) measured in tethered cell assays coupled to flagellar filament polymorphic transitions could be correlated with the swim-tumble motility of free-swimming bacteria. This literature has been reviewed [3]. It established there was a fundamental difference in switch design and operation between bacterial and eukaryotic flagella (see [4] for a minireview). Advances in bacterial flagellar switch function and structure in the second half of these thirty years were based on the development of high-throughput genetic screens, sophisticated motor rotation assays, isolation and biochemical characterization of the intact switch and sub-complexes together with atomic structures as summarized in this section (Figure 1). Subsequent sections in this review consider progress in switch dynamics and architecture in the light of these advances. 

*The structural complexity of the flagellar switch:* Swarm plate assays [5] provided high-throughput isolation of motility mutants that could be grouped into three categories (non-flagellate (*fla*), non-motile (*mot*) and non-chemotactic (*che*)). In 1986, a switch complex of three interacting proteins was proposed based on swarm plate assays of suppressor mutations [6]. Five years later, the gene sequences encoding the proteins were obtained by polymerase chain reaction (PCR), an early example of the use of PCR in bacterial motility research. The sequences indicated that all three proteins (renamed FliG, FliM and FliN) were cytoplasmic [7], and revealed mutation hotspots. The *che* mutations mostly localized to *fliM*; the *mot* mutations largely localized to *fliG* [8,9]. The clustering of the *fliM che* mutations to distinct regions that accentuated CW or CCW rotation suggested that the FliM structural determinants assigned the rotation state [8]. Genetic evidence for electrostatic residue interactions between the FliG C-terminal and MotA [10,11] implicated FliG in motor function. The *motA* and *motB* genes only had *mot* alleles in contrast to genes for the switch complex. Tethered cell motility was resurrected in similar, stepwise increments by *motA* or *motB* induction in the corresponding deletion strains, implying multiple, independently acting MotA and MotB stator complexes [12]. None of the protonatable *E. coli* FliG, FliM or FliN residues that were sites for *mot* substitutions was essential for motility [13]. These data indicated that the energizing proton flux did not traverse the switch complex.

The development of gentler protocols in 1992 led to the isolation and morphological identification of the switch complex, based on its impaired mutant structures, as an extended cytoplasmic component of the basal body [14], subsequently termed the C ring [15]. Further purification enabled its biochemical characterization [16]. FliN copy numbers (n) were 3–4 times the estimates for FliM (n = 34 ± 3). Analysis of the sub-complexes concurrently established that the switch proteins self-associate and interact with each other [17]. Finally, structures that were morphologically identical to the C ring formed upon overproduction of the switch complex proteins together with the FliF MS ring [18]. 3D reconstructions in ice of the *S. enterica* basal body [15] combined developments in cryo-electron microscopy with single-particle image analysis (reviewed in [19,20]) to resolve C ring periodicity [21] and position individual domains, with FliG an early example [22]. The overproduced C rings had variable symmetry (n = 32–38) [23], but the dominant 34-fold symmetry was consistent with the biochemical estimates of FliM copies in the native C ring. This advance exploited the fact that *fliF*, *fliG, fliM* and *fliN* were “early” genes in the flagellar regulon [24], and built upon MS ring assembly by FliF overproduction [25].

*Torque generation and switch activation:* The torque, T, on a rotating spherical tethered cell of radius, a, is balanced by the hydrodynamic drag. 8Πηa^3^W, where W is the angular velocity, and η the medium viscosity. 

The torque velocity relation was examined over a limited load (8Πηa^3^) range by changing η [26]. The relation had a biphasic form for CCW rotation. Visualization of the rotation of the “tethered” beads attached to flagellar stubs extended the range of the torque velocity relation to high rotation speeds. The torque was constant at low speed, and then decreased linearly above a threshold speed. Temperature and isotope effects in the linearly decreasing, but not the constant, torque regime implied the energizing proton transfer reactions limited the decreasing velocity [27]. Comparable results were obtained for the sodium *Vibrio alginolyticus* motor [28]. Application of an external force with optical traps [29] or electrorotation [30] allowed the study of the relation at negative as well as positive torque. The integration of optical trap and bead rotation assays revealed the load-dependent modulation of CW rotation interval [31], showing that the switching mechanism was not isolated from motor mechanics. Rapid switching events, damped out in tethered cells due to compliance of the hook structure connecting the basal body with the flagellar filament, were resolved earlier by laser dark-field microscopy measurements of filament rotation under conditions similar to free-swimming bacteria [32]. This study recorded slowed rotation and pausing events in addition to rapid reversals and importantly showed that these events increased in strains carrying switch complex mutations providing a direct window into switch mechanics not accessed by swarm plate assays. 

The atomic structure of the FliG carboxy-terminal domain (FliG_C_) [33] heralded the molecular era in structural analysis of the switch complex. Armadillo (ARM) folds, a ubiquitous architecture found in signal proteins, characterized both middle (FliG_M_) and C-terminal FliG domains [34]. The structures revealed that charged FliG residues essential for torque generation [11] localized to a surface-exposed face of an α-helix. Suppressor residue substitutions for the MotB stator protein [35] clustered to the FliG_M_–FliG_C_ inter-domain loop that included a conserved glycine pair. The CW and CCW biasing substitutions mainly localized to FliG_M_ and the inter-domain linker, but importantly also to the FliG_C_ conserved MXVF loop and adjacent α-helices. 

*Ultra-sensitivity of the chemotactic motor response:* The 1989 atomic structure of the chemotaxis signal protein CheY [36] identified a cluster of three aspartate residues as the probable phosphorylation site. This study was an early application of site-specific mutagenesis for structure determination in bacterial motility and chemotaxis. Subsequent studies established that CheY aspartyl phosphorylation by the receptor associated CheA kinase coupled receptor occupancy to the motor response. CheY is one of a large superfamily of response regulators with diverse functional roles (reviewed in [37]). Aspartyl phosphate is labile in contrast to the corresponding serine/threonine phosphates exploited in developmental circuits, but beryllium fluoride (BeF3), an acyl-phosphate analogue, binds stably [38]. Biochemical studies determined the FliM N-terminus (FliM_N_) to be the CheY binding target at the flagellar switch [39,40]. Phospho-CheY and BeF3-CheY had a comparable affinity, with the activating structural transitions visualized in the BeF3-CheY.FliM_N_ crystal structure [41]. 2D-NMR further showed the bound FliM_N_ influenced phosphorylation site dynamics [42]. CheY did not associate with incomplete switch complexes formed by FliF MS rings with FliG [43]. A library of *cheY* mutant alleles was generated guided by the atomic structures (reviewed in [37]). The phospho-mimetic mutations 13DK and 13DK106YW have figured prominently in the study of switch physiology (e.g., [44]).

The motor rotation bias, reported by tethered beads, was a function of intracellular GFP-tagged CheY concentration, estimated by correlation intensity analysis in single *S. typhimurium* cells. The bacteria carried mutations that ensured CheY was phosphorylated. The bias changed sharply with CheY concentration (Hill coefficient, *H* = 10.3) [45]. Binding assays of CheY with overproduced complexes reported a similar difference in affinity for the phosphorylated and non-phosphorylated forms to that for FliM_N_, but the binding was not cooperative (*H* ~ 1) [46]. Early models had formalized the switch as an equilibrium thermal isomerization machine [44,47]. An important advance over these models was the conformational spread model. that explicitly considered the multiple subunit stoichiometry, *N*. The individual subunits fluctuated between the CW and CCW states with adjacent subunits linked by a coupling energy term influenced by ligand (CheY) occupancy. The mean size of the contiguous CW or CCW domains increased with the coupling energy. Above a critical threshold, the entire ring flipped as a 1D Ising type switch to simulate the ultra-sensitive response with n = 34 [48].

In conclusion, a cluster of key publications between 1986 and 2003 (A) established a conceptual framework for the bacterial flagellar motor (BFM) switch and (B) introduced new methodologies pivotal for future advances in structure and dynamics. (A) The idea of the switch had developed from a process, rotation reversal, to a material entity, the switch complex, to a physical object, the C ring, The C ring was composed of a small set of core proteins that self-assembled into a large multi-subunit assembly attached to the MS-ring. The C ring did not conduct protons but interacted as the rotor module with the proton-conducting Mot complexes to generate torque. Mutant phenotypes linked component lesions to switch phenotypes, influenced by motor operation, that included, but were not restricted to, rotation reversal. The switch set-point was shown to be an ultrasensitive function of the activity of the CheY signal protein, in contrast to non-cooperative CheY binding to the C ring. The linkage between the highly cooperative output (motor rotation) and the non-cooperative input required long-range allosteric communication across subunits between the FliM_N_ binding sites for CheY and the FliG_C_ interface with stator complexes. A formal model was developed [48] while atomic protein structures [33,34,36,41] provided important clues into possible interactions. (B) The period witnessed the timely application of new NMR methodologies for structure determination of large macromolecules [49] to CheY complexes [42], as well as cryo-EM allied single-particle image processing of multi-subunit assemblies [19,20] to isolated basal bodies [15,23,50]. The *Thermatoga maritama* FliG structures [33,34] set an important precedent for X-ray crystallography of thermophile switch proteins. The first applications of live cell imaging with GFP biotechnology [51] to determine CheY bias modulation [45] or localization [43]—and together with single-molecule, force microscopy to characterize the load-dependent switching [31]—were to prove equally influential. 

Four fundamental issues could now be addressed. First, what was the nature of the coupling between the FliG_C_ motor domain and the FliM_N_ CheY binding target? Secondly, how were the dynamics of the C ring, a large multi-subunit assembly, synchronized for smooth rotation and rapid reversal? Thirdly, how did CheY activation trigger an ultrasensitive switch response? Fourthly, how was the switch regulated by the motor operation as dictated by the torque–velocity relations?

## 2. Switch Physiology and Mathematical Models

**Figure 2:**
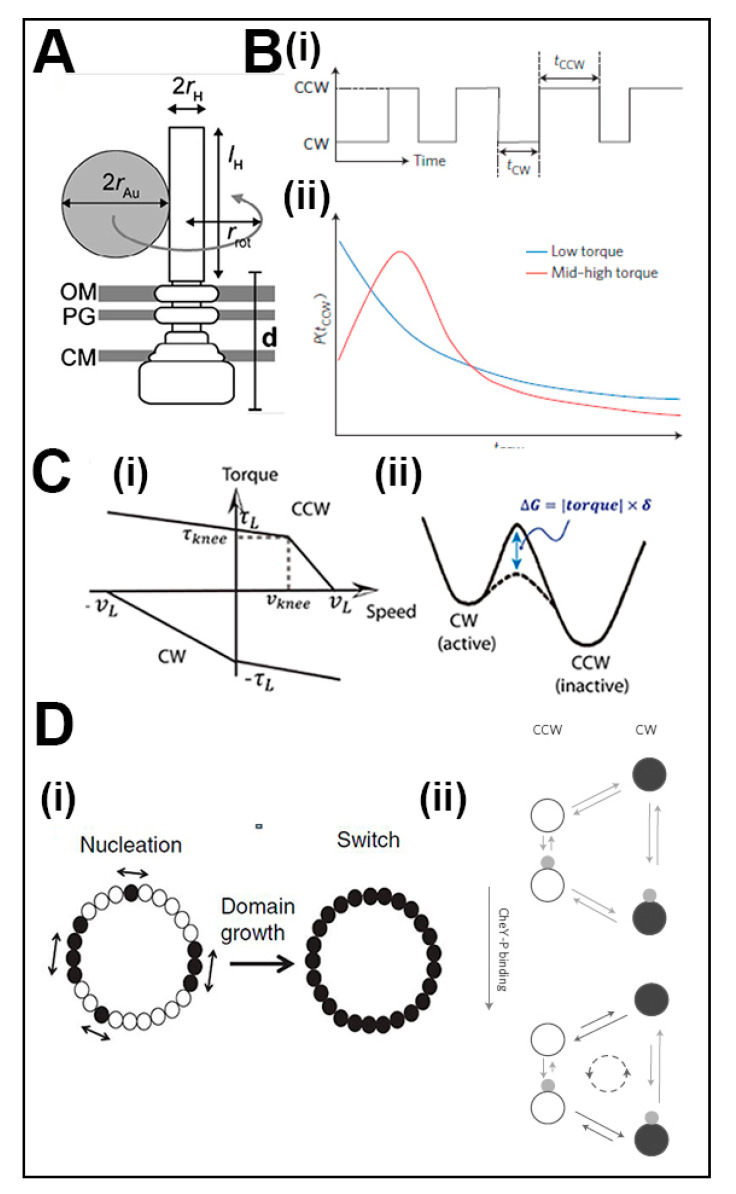
Advances in switch physiology. (**A**) Time-resolved motor rotation: Schematic of a motor rotation assay with a nanosphere conjugated to the hook connector contiguous with the rod and basal body (reproduced from [54] with permission). (**B**) CW and CCW interval distributions: (**i**) Idealized time series of a motor alternating between CW and CCW rotation. (**ii**) Interval (τ_CW_, τ_CCW_) distributions measured under low and high torque (reproduced from [75] with permission). (**C**) Coupled energization and switching of rotation: (**i**) Torque velocity curves for CW and CCW rotation [63]. (**ii**) Free energy diagram of CW <-> CCW transitions and the mechanical work (blue arrow) contribution (reproduced from [72] with permission). (**D**) Models for non-Poisson interval distributions: (**i**) Conformational spread seeded from multiple CheY binding events (reproduced from [59] with permission). (**ii**) Breakdown of detailed balance from motor energy dissipation (reproduced from [64] with permission).

Motor dynamics are the direct outcome of the architectural dynamics of the molecular machinery. Knowledge of motor dynamics, and the associated development of mathematical models, has advanced concurrently with knowledge of the molecular architecture and dynamics. The spatiotemporal resolution and mechanical range of the rotation assays have continued to increase for characterization of speed fluctuations, the load dependence and stochastic properties of the switch machinery in unprecedented detail. These advances provide additional constraints that must be addressed by the study of the molecular mechanism. Motor dynamics have been reviewed recently [52]. An overview is given in this short section, summarized in Figure 2, to provide additional context for the main body of the review. 

Focal back-plane interferometry with high spatiotemporal resolution (1°, 10^−3^ s) recorded incomplete, intermediate switching events to extend the temporally resolved measurement of switch transitions [53]. More recently, gold nanospheres (d = 60 nm) conjugated to genetically engineered, rigid *Salmonella* flagellar hooks, imaged by dark-field and rapid (5000 Hz) CMOS cameras, have been exploited to measure motor rotation [54]. This study interpreted the large speed fluctuations recorded near the zero-torque speed (~400 Hz) that persisted over many revolutions as the association–dissociation of individual stator units. The highlight of such studies was that the resolution of the angular step periodicity per revolution was resolved in motors resurrected by single or a few stator units; first for a sodium powered chimeric motor [55] and then for the *S. enterica* proton motor [56]. In each case, 26 steps per revolution were reported for both CW and CCW rotation. However, while the step size was symmetrical for the *Salmonella* motor, the CW steps were smaller than the CCW steps in the chimeric motor. A study of the unidirectional *R. sphaeroides* motor reported a similar estimate of 27–28 discrete stopping angles [57]. The steps may be due to modulation of the elastic potential well by periodic contacts between the static and mobile motor modules rather than individual energy coupling events. A formal model of this idea successfully predicted the difference in CW versus CW step size [58].

The long-standing tethered cell measurements of the Poisson-distributed CCW and CW intervals had been the bedrock for theoretical models. Filament-associated bead rotation measurements in the phospho-mimetic *E. coli* CheY13DK mutant strain first revealed gamma distributions for both CW and CCW intervals [59] to challenge the view of switch transitions as a single Poisson process based on tethered cell data. Subsequent measurements of the rotation of beads attached to flagellar stubs [53] and nanoparticles attached to flagellar hooks [60] obtained exponential distributions in apparent contrast to [59], fueling speculation that filament polymorphic transitions [61] may have complicated the rotation of filament-associated latex beads (0.5 μm). The use of different-sized latex spheres extended the dependence of switching kinetics on load [62]. The study reported that at near zero-load speed both CCW<->CW and CW<->CCW transitions increased with the load. Furthermore, the torque–velocity curve for CW rotation was linear in contrast to CCW rotation [63]. The load dependence raised the possibility that it may underlie the apparent discrepancy between the gamma and Poisson interval distributions. The comprehensive analysis of the rotation of different-sized beads in the double mutant phospho-mimetic strain CheY13DK106YW showed this was indeed the case. Both exponential and peaked distributions were obtained, depending on the different load, proton potential and torque [64], to rule out speculation that the gamma distribution was an artefact. 

The knowledge about the regulation of (CW/CCW) rotation bias by CheY has also advanced. Temporally resolved recordings of bead rotation over minutes re-evaluated motor individuality in terms of its rotation bias. Long-term recordings of variation in single bead rotation compared to population variation of multiple beads concluded that individuality was due to intracellular chemistry. The bias of individual motors had a bimodal distribution with cells with high switching frequencies binned into a central trough. The CW and CCW fractions were dominant, consistent with the ultrasensitive switch in rotation bias by CheY [65]. Single bead CW and CCW rotational intervals had earlier been reported to be asymmetrically distributed around the bias midpoint ((CW/(CW + CCW)) = 0.5), with the CCW but not CW intervals varying with rotation bias [66]. These different relations argued against the determination of transition rates by CheY or any other single parameter. The ultra-sensitivity has also been re-evaluated. The local GFP-CheY concentration around single flagellar motors was measured and correlated with (CW/CCW) rotation bias in a ∆*cheY E. coli* strain [67] for an estimate of the mean CheY rotor occupancy during CW rotation. GFP-tagged fusion proteins also provided evidence that the basal C ring components FliM and FliN undergo dynamic exchange [68,69]. An experiment was designed to correct for the FliM copy variation that would be obtained based on the exchange being adaptive. It estimated the Hill coefficient, *H*, for the ultra-sensitivity from bias changes due to attractant removal and addition in strains lacking receptor adaptation was as high as 21 [70].

The experiments motivated the development of the conformational spread model [48] and the formulation of a fundamentally distinct non-equilibrium model [71]. The initial interpretation for the peaked gamma distribution was based on multiple Poisson events [59] to refine, but not radically alter the conformational spread model. The asymmetry in the experimental torque–velocity curves and the load dependence of the switching kinetics could also be accounted for by an integrated model that combined torque generation models with the switch ultra-sensitivity as formalized by conformational spread [72]. Theoretical models, in general, seek to explain the interdependence between rotation bias, switching frequency and interval distributions. The experimental relations between these motor parameters were set as constraints to model the coupling between synchronous switching and signal amplification as a function of subunit number [73]. More recent simulations explained the correlation between local CheY concentration and single motor rotation bias as well as the utility of dynamic subunit exchange in the maintenance of switch ultra-sensitivity [74]. Detailed balance within the equilibrium conformational spread framework was followed in both studies [73,74]. Tu’s non-equilibrium model, where the detailed balance is broken based on energy input, also explains the gamma distribution data [71,75]. The model is consistent with the expanded set of interval distributions obtained under different load and energization conditions as detailed in [64]. The energy input for switching need only be a small fraction of the total input that is dominantly utilized to power rotation. 

## 3. Architecture and Molecular Mechanism

Structural work on the issues outlined in the previous two sections can be grouped into four major areas based on the protein–protein interactions: the central processing unit (FliM_M_.FliG_N_), the trigger machinery (CheY/C ring), the torque reversal mechanism (FliG_C_/FliG_M_) and the torque transmission platform (FliG_N_/FliF_C_). 

### 3.1. The Central Processing Unit—The FliM_M_.FliG_M_ Complex

**Figure 3:**
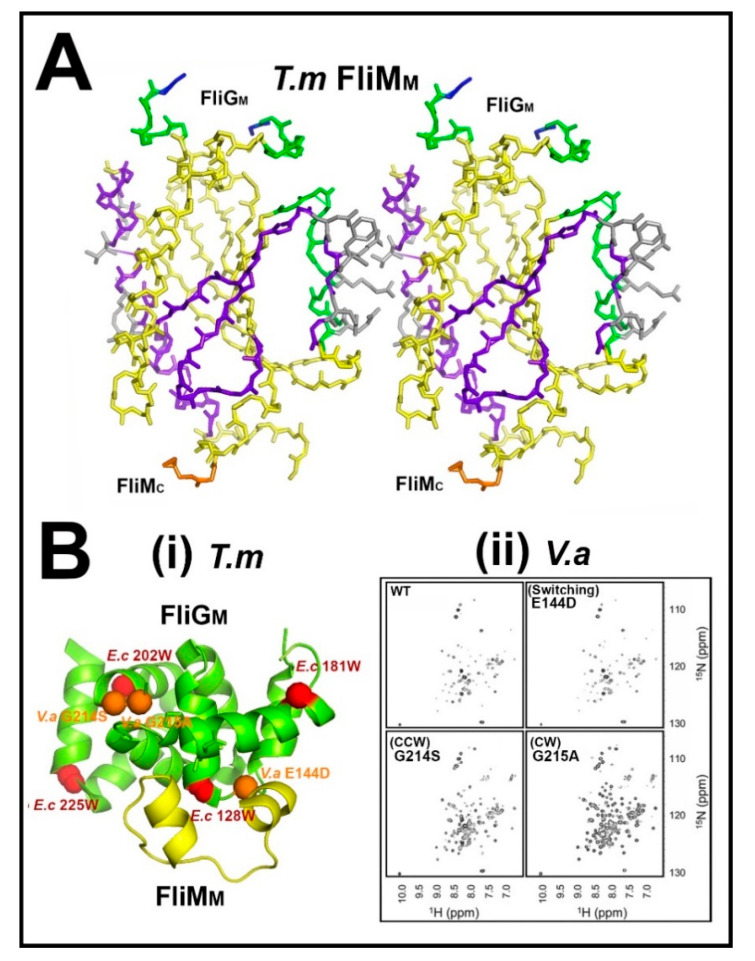
The central signal processing unit. (**A**) FliM_M_ dimer model. The model is based on the *T. maritima* FliM_M_ structure (gold)) guided by in situ cross-link data (grey). Localized CW (green) and CCW (magenta) biasing residue substitutions from bacterial homologs are mapped onto the colored structural elements. FliG GXXG interaction loop (blue). C-terminus (orange) (PDB: 2hp7 (modified from ([76])). (**B**) The central processing interface—FliG_M_-FliM_M_. (**i**) *T. maritima* structure (PDB: 4fhr) highlighted with homologous FliG_M_ residues to those implicated by *E. coli* tryptophan mutagenesis (red [82]) and *V. alginolyticus* switch mutants G214S, G215A and E144D (orange [83]). (**ii**) 2D-NMR ^1^H–^15^N correlation spectra reveal that the native (WT) *V. alginolyticus* FliG_M_ architecture is substantially altered by substitutions in the conserved glycine pair that alter rotation bias, but is unaffected by the EPQR E144D residue substitution that increases the switching frequency. Axes indicate the shift in the magnetic field, in parts per million (ppm), relative to a reference compound for resonance (reproduced from [83] with permission).

X-ray crystallography led the effort guided by mutagenesis, in situ crosslinking and EM reconstruction to characterize the linkage between the domains responsible for torque generation and CheY signal reception. The atomic structure of the *T. maritima* FliG middle (FliG_M_) and C-terminal (FliG_C_) domains had an established ARM architecture for both domains and localized hotspots for *E. coli che* mutations to the sequence encoding the linker region between FliG_M_ and FliG_C_ [34]. The atomic structure of the *T. maritima* FliM middle domain (FliM_M_), the central element in switch function, showed that the CCW and CW substitutions localized to residue positions at the intradomain contact interface, as deduced by subunit crosslinking in situ [76]. The CW substitutions aligned differently to the CCW substitutions, suggesting distinct FliM_M_ orientations for CW versus CCW rotation (Figure 3A). In situ crosslinking had been introduced in earlier work on FliG_M_ domain organization [77]. The in-situ crosslinking experiments probed the subunit organization of the switch proteins as appropriate controls established that cross-linking was negligible in non-flagellate strains [76,77]. The structures of FliM_M_.FliG_M_ complexes from *T. maritima* [78,79] and *H. pylori* [80] determined the functional importance of the FliM GXXG and FliG EHPQR loop motifs at the FliM_M_.FliG_M_ interface. Comparison of the *T. maritima* and *H. pylori* interfaces showed conservation of essential GXXG and EHPQR loop contacts and their stabilization by complex formation. Additional interfacial residues important for coupling were identified in *H. pylori*, supported by mutagenesis.

Protein dynamic simulations showed that the core architecture and dynamics of FliM_M_ do not change upon complex formation and are conserved between *T. maritima* and *H. pylori.* FliM_M_ conformational ensembles generated from the *T. maritima* structure had a bimodal distribution, while the FliG_M_ domain movements also alternated between the bi-stable states strongly coupled to FliM_M_ [81]. Targeted tryptophan substitutions had identified FliG_M_ residue positions important for association with FliM_M_ [82]. In addition to the EHPQR loop, these residues were in a FliGc ARM-C hydrophobic patch adjacent to the FliG_MC_ GG linker as well as non-interfacial FliG_M_ residue positions. Recent NMR measurements of the FliG_M_ dynamics in the sodium *V. alginolyticus* motor have provided important validation, supported by mutagenesis, of the conformational plasticity of the FliG_M_ domain between the bi-stable conformational states [83]. The EHPQR E144D residue substitution increased the switching frequency, a similar response to one obtained upon phenol repellent addition. It did not alter FliG_M_ conformation. In contrast, residue substitutions in the GG hinge both locked the bias and reduced the FliG_M_ dynamics, as assessed by NMR. The E144D and CCW-biased G214S motors were both CCW-locked in the ∆*che* strains, implying a close linkage between CheY occupancy and FliG_M_ conformational fluctuations (Figure 3B). 

In *H. pylori*, co-crystallization of putative spermidine synthase (SpeE) with FliM_M_, combined with mutagenesis and motility assays, has shown how this protein affects motile speed and switching behavior. The SpeE.FliM_N_ contact interface partially overlaps with the FliG_M_.FliM_M_ interface [84]. While several proteins important for cell metabolism have been identified in diverse bacteria to influence motile behavior via direct interaction with FliG (cited in [84]), SpeE is the first known to act at the FliG_M_.FliM_M_ interface. In conclusion, the crystal structure, allelic mutations, in situ crosslinking, and protein dynamic simulations all argue for the alternation of FliM_M_ between bi-stable conformational states, inter-species conservation of its fold and interfacial FliG_M_ coupling.

### 3.2. The Trigger Machinery—CheY and the Basal C Ring

Motor response to chemotactic signals can be parsed into CheY activation and binding to the C ring to bias FliM_M_ conformational fluctuations. There is a 20-fold increase upon phosphorylation in CheY affinity for *E. coli* FliM_N_ [41]. The molecular analysis of CheY activation by the phospho-mimetic CheY 13DK106YW double residue substitutions used to study motor response was initiated by co-crystallization of the *E. coli* protein alone and in complex with the FliM N-terminal peptide (FliM_N_) [85]. Strikingly, while the CheY13DK106YW in the complex was in a conformation akin to BeF3 activated CheY, the free CheY13DK106YW adopted the inactive conformation. Subsequently, the atomic structure of the native CheY.FliM_N_ complex revealed that the electron density of a critical hinge, the α4–β4 loop, in this complex partitioned between its active and inactive states [86]. Quantitative comparison of MD conformational ensembles from crystal structures of the CheY superfamily representatives has extracted common signatures, including the α4–β4 hinge, for allosteric communication between the phosphorylation and target binding sites [87]; the importance of the α4–β4 loop further emphasized by MD simulations of acetate-activated CheY [88]. MD simulations supported by oxygen-radical foot-printing solution measurements, a sensitive probe for sidechain solvent accessibility [89], have now shown that closure of this loop hinge buried the allosteric relay aromatic sidechains to both stabilize the global CheY fold and increase the local FliM_N_ affinity. 

**Figure 4:**
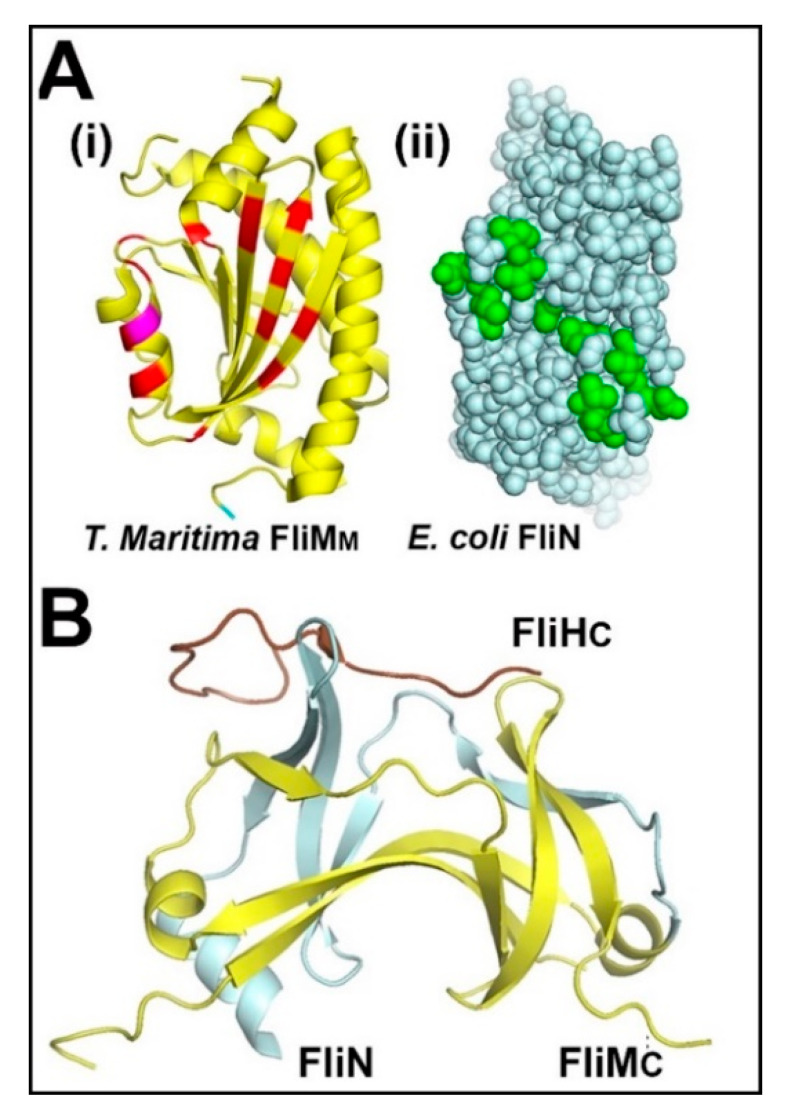
Chemotactic signal processing in the basal C ring. (**A**) Secondary CheY binding sites: (**i**) *T. maritima* FliM_M_ residues showing NMR chemical shifts (red) in the presence of BeF3-activated CheY. Strongly shifted residue position (magenta) (PDB: 2hp7 (modified from [90])). (**ii**) *E. coli* FliN homodimer showing residues that impair binding of an activated CheY-FliM_N_ fusion protein. Substitutions at these residue positions result in CCW rotation (PDB: 1yab (modified from [91])). (**B**) Basal C ring architecture: (**i**) Atomic structure of the FliM_C_ (yellow)–FliN (cyan) heterodimer in complex with the FliHc terminal peptide (brown) fused with lysozyme (PDB: 4yxc (modified from [94])).

The motor response is likely to depend on the intervening disordered linker between FliM_N_ and FliM_M_ that may, in principle, serve as a flexible tether to deliver FliM_N_-bound CheY to second binding sites. Occupancy of these sites could control FliM_M_ conformational fluctuations and inter-subunit coupling. Candidates have been identified in two species. There is NMR evidence for an interaction between *T. maritima* FliM_N_-tethered CheY and FliM_M_ [90]. Alternatively, in *E. coli*, pull-down assays have reported evidence for an association of the CheY-FliM_N_ fusion with FliN [91]. This evidence is summarized in Figure 4A.

There is a close homology between FliN and FliM_C_. The atomic structure of the *T. maritima* FliN homodimer [92] motivated mutagenesis and in situ crosslinking experiments to infer FliN tetrameric quaternary organization in the *E. coli* flagellar motor [93]. These experiments reported cross-link changes upon addition of chemotactic stimuli, consistent with domain motions, but the connectivity between FliM_N_ and the FliM_C_^1^.FliN^3^ module is not well-understood as the FliM inter-domain linkers have not been structurally characterized. The atomic structure of the FliM_C_.FliN heterodimer is similar to the FliN homodimer [94], consistent with a FliM_C_^1^.FliN^3^ tetramer identified by mass spectroscopy and structural homology with the Type III injectosome components [95]. The C-terminal tail of the FliH component of the flagellar export ATPase assembly has been co-crystallized and bound to the FliM_C_.FliN heterodimer (Figure 4B). The co-crystal validated early predictions of the interactions of the C ring with the flagellar export apparatus [96], as well as the proposal based on cryo-tomographic (cryo-ET) reconstruction and FliH sequences from diverse species that FliH acts as a spacer to set C ring diameter [97].

The switch complex contains FliY instead of, or in addition to, FliM and FliN in numerous species [98]. The structure of *T. maritima* FliY reveals a middle domain with strong structural similarity to FliM_M_, while the FliY C-terminal domain is similar to FliN [98]. This study also reported solution assays to show that *T. maritima* FliY homodimerizes via its N-terminal domain and does not have an increased affinity for activated versus inactive CheY, and that its middle domain does not bind FliG. FliY was first reported in the Gram-positive *B. subtilis* [99] where CheY phosphorylation enhances CCW, not CW rotation [100], in contrast to the Gram-negative γ-proteobacteria *E. coli* and *S. enterica*. The characterization of the *B. subtilis* rotor module by in situ crosslinking found that the FliM_M_FliG_M_ interface is conserved as in other bacteria with FliY proposed to form an external ring adjacent to the FliM_M_ ring [101].

Thus, FliM_C_ interacts with FliN to form the C ring base and can, in principle, transmit structural perturbations triggered by CheY-FliN association to FliM_M_. Other bacteria, such as *T. maritima*, may utilize a different signal strategy and a distinct basal ring architecture. 

### 3.3. Bidirectional Torque Generation—FliG_M_–FliG_C_ Interactions

Vital clues driven by FliG multi-domain crystal structures have emerged since 2003 on the mechanism of rotation reversal, albeit from a limited set of organisms (*S. enterica*, *T. maritima*, *H. pylori* and *A. aeolicus*). Mutagenesis screens, experimental and computational analyses of protein dynamics and coevolutionary information have supplemented the crystal structures in important ways, as outlined below. 

The variable orientations of the FliG_C_ domain relative to FliG_M_ inform on both rotation reversal in addition to assembly. A striking example has been a pair of *H. pylori* FliG_MC_ structures that show FliG_C_ in orthogonal orientations relative to FliG_M_ [102]. The underlying motions have been characterized by experimental probes and atomistic protein dynamics simulations since the snapshots of the kinetic mechanism provided by the crystal structures, though valuable, are too sparse to determine reaction trajectories. FliG_C_ reorientation was first deduced from in situ crosslinking in *E. coli* [77]. Reorientation of the *S. enterica* α-helical linker (helix_MC_) adjacent to the GG pair was inferred from in vivo crosslinking experiments and simulations, to regulate the switching between the CW and CCW states. The crosslinking experiments found that the helix_MC_ G174C residue substitution formed crosslinks in the CW-locked FliG_PEV_ but not the native, dominantly CCW *S. enterica* strain [103]. Analyses of conformational ensembles simulated from the FliG_MC_ structures had identified helix_MC_ as a central hinge and predicted its melting regulated the FliG_C_ orientation relative to FliG_MC_ [81]. These studies, taken together, supported the large reorientations deduced from the superimposition of different crystal structures. 

**Figure 5:**
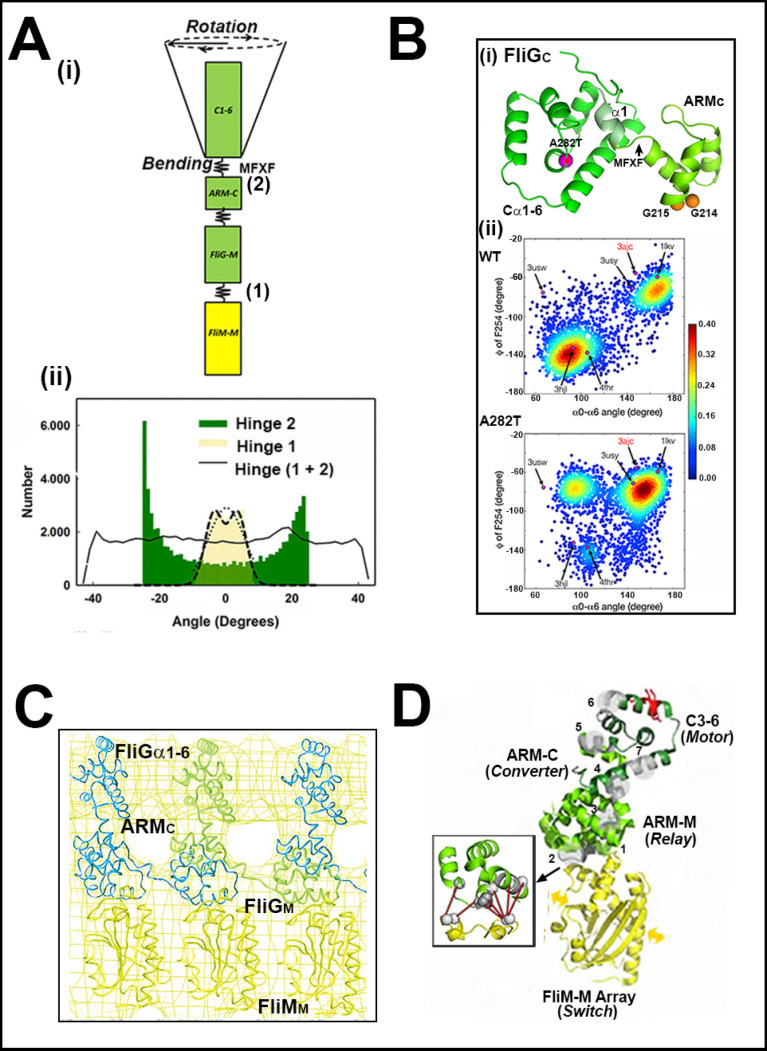
Mechanics of rotation reversal. (**A**) Mechanical amplification: (**i**) The *T. maritima* FliM_M_FliG_MC_ crystal structure (PDB: 4fhr. FliM_M_ (gold), FliG_MC_ (green)), modelled as a segmented rod with flexible hinges. Two-stage amplification of FliM_M_ motions triggers large FliG_C_ domain reorientations. (**ii**) Bimodal hinge planar angle distributions from the dominant principal collective motions 1–3 extracted from the generated conformational ensemble. Lines plot the 3D angular displacement distribution (solid) and unimodal fit (dotted). The distribution of the FlIM_M_FliG_M_ interfacial hinge (1 (gold)) has a narrow spread relative to the MFXF hinge distribution (2 (green)), indicating most of the amplification occurs at the latter hinge (from [81]). (**B**) MFXF hinge dynamics: (**i**) The representative structure of the *V. alginolyticus* FliGc A282T homology model from cluster analysis of the MD ensemble. The predicted dynamic helix α_1_ (pale green), the MFXF_254_ motif, and residues G214, G215 (orange) and T282 (magenta with an asterisk) are marked. 2D-NMR reports the CW-biasing A282T residue substitution melts helix α_1_ and reorients MFXF_254_. (**ii**) The 2D plot of F254 dihedral angle versus helix α_6_ orientation relative to the FliG_M._FliG_C_ interface obtained from the MD trajectories. The wild-type (WT) and mutant (A282T) conformational clusters map onto different subsets of the FliGc crystal structures. Labeled circles (magenta) mark structures from *T. maritima* (CW-locked. PDB: 3ajc; wild type. PDB: 1lkv; complexed with FliM_M_ (PDB: 4fhr)), *Helicobacter pylori* (PDB: 3usy and 3usw) and *A. aeolicus* (full-length. PDB: 3hjl) (from [113] with permission). (**C**) The FliG_M_-ARMc stacking interaction: The best-fit of the pulsed dipolar ESR spectroscopy model of the *T. maritima* FliG_M_-ARMc inter-domain stack to the 3D *S. enterica* electron density map (from [108] with permission). (**D**) Residue coevolution model for rotation reversal: Strong, coevolved FliM_M_ (yellow) contacts mediate conformational spread (arrows) in the FliM_M_ ring. The FliG αC3-6 “motor domain” (dark green) is organized around the αC5 “torque helix” with charged residues (red) important for rotor–stator interactions. The primary nodes of the coevolved network form a relay of allosteric sectors (numbered grey patches) across ARM-M and ARM-C (light green). ARM-C has sparse coevolved contacts implying a variable fold to generate different motor responses from a conserved FliM_M_ switch transition Box. Coevolved interfacial pairs (residues (white spheres) and contacts (red)) link the FliM_M_ GGXG motif with the FliG_M_ EHPQ motif. The contacts and nodes are mapped onto PDB: 4fhr (from [110]).

The simulations also reported that the *T. maritima* MFXF linker between FliG_C_ ARM-C and Cα_1-6_ amplifies thermal fluctuations of the coupled FliM_M_–FliG_M_ central processing unit [81]. This idea was supported and extended in important ways by NMR and MD simulations of *V. alginolyticus* FliG_C_. The conformational dynamics of FliG_C_-Cα_1-6_ helix α1 in FliG A282T were reduced relative to the native protein to give detectible peaks in the NMR spectra. The A282T strain has a CW bias in contrast to the CCW bias of the native strain. MD simulations further showed the reduction was due to additional hydrogen bonding contacts between the ARM-C and FliG_C_ α1-6 that constrained the flexibility of the intervening linker. MFXF_254_ hinge orientation, monitored by residue F254, partitioned into conformational clusters that overlapped the subsets of the crystal structures representing either the CW or CCW rotation states based on other criteria (Figure 5A,B). A prescient early analysis of *E. coli* FliG residue substitutions in the helix_MC_ linker, sensitized by a serendipitous E232G substitution a few positions upstream of the MFXF hinge, had reported these gave rise to diverse phenotypes with altered switching frequencies, pausing or altered bias [104].

The complete *Aquifex aeolicus* FliG structure revealed an ARM fold for the N-terminal domain (FliG_N_) in addition to FliG_M_ and FliG_C,_ separated by α-helical linkers [105]). Sequence similarity suggests these domains arose from gene duplication [106]. The structure reported inter-molecular stacking between the FliG_M_ and FliG ARM_C domains. In contrast, the *S. enterica* FliG_PEV_ [107] and *T. maritima* [78] crystal structures of the FliG_MC_ complexes showed intramolecular stacking of these domains. The consensus view now is that the assembly of the FliG middle and C-terminal domain is mediated by intermolecular stacking. Solution pulsed dipolar ESR spectroscopy combined with residue substitutions first indicated that, in *T. maritima*, these domains self-assemble via the FliG_M_–FliG ARM_C intermolecular stacking contact; then, direct assembly of FliM [108]. The central and membrane-proximal sections of the *S. enterica* C ring map was fit well by the ESR-derived model (Figure 5C). A domain-swap mechanism for FliG ring assembly in the *E. coli* motor was subsequently determined with SAXS analysis supported by in situ cross-linking [109]. Binding energy from FliG association with the FliF MS ring was speculated to alter the conformational equilibrium between the compact and extended conformation of the malleable helix_MC_ to favor polymerization of a chained FliG_MC_ ring in the flagellar motor but not in solution. 

Coevolutionary information has emerged as an important high throughput tool to assess the design principles of bacterial multi-protein complexes at the single residue level. Residue coevolution supported the design of the switch machinery framed by experiments and simulations. First, the role of FliM_M_ as the central relay was consistent with its strongly coevolved inter-subunit and FliG_M_ interfacial contacts [110]. Two distinct *T. maritima* FliM_M_ dimer configurations were obtained when dimer formation was simulated based on coevolved subunit couplings [111], although one orientation did not match either the CW and CCW dimers deduced from *S. enterica* residue substitutions [76], either because of limited sampling and/or because the bi-directional switch is not universal across species. Secondly, coevolution provided strong support for conservation of the FliG_C_–FliG_M_ stacking contact [109,112] relative to other contacts identified by cross-link data [77]. Thirdly, the coevolved coupling was mapped, in part, onto the identified dynamic couplings and modules. Notably, the coevolved FliM_Mc_–FliG_M_ contacts was mapped onto the dynamic couplings across the *T. maritima* FliM_M_.FliG_M_ interface [81] and FliG_MC_, as a coevolved network with distinct nodes as allosteric sectors [110]. The nodes included the well-characterized EHPQR and PEV (in *S. enterica*) at the FliM_M_.FliG_M_ interface as well as α-helices (helix_MC,_ Cα_1-6_ helix α_1_). The melting of these α-helices has been noted above. The coevolution signal from FliG_C_ ARM-C is sparse. This sub-domain may be the converter element that encodes different species-specific outputs from a conserved input signal (Figure 5D).

In conclusion, the library of crystal structures now available, together with experimental and computational measurements of their dynamics, position FliM, FliG and FliN in the *S. enterica* C ring map and charts out their connectivity and conformational plasticity. FliM_M_ has a central location in the C ring with subunit contacts designed for propagation of both the CW and CCW conformational states in the species studied thus far. FliM_M_ forms a tightly coupled complex with FliG_M_, with a central location in the C ring, connected via FliG_M_ to FliG_C_ adjacent to the membrane stator complexes, and via FliM_M_ to FliM_C_ in the basal C ring.

### 3.4. The Association of the Switch with the Mot Stators and the FliF_C_ Scaffold

**Figure 6:**
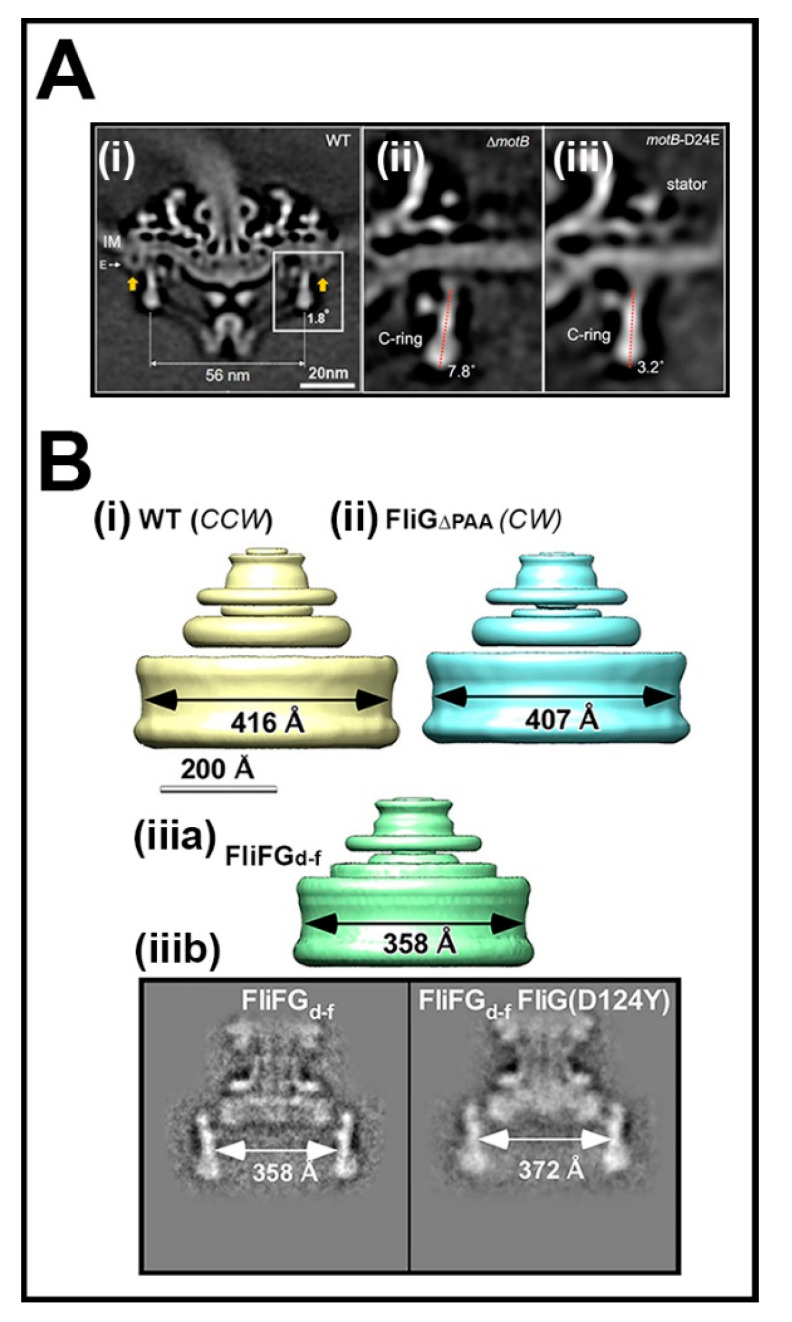
C ring modulation by interfacial membrane protein interactions. (**A**) C ring modulation by stator operation: (**i**) Central tomogram section of the wild-type *B. burgdorferi* flagellar basal structure. The C ring wall (box) is almost perpendicular (1.8°) to the Mot stator complexes (yellow arrows). The C ring has different orientations in (**ii**) ∆MotB (7.8°) and (**iii**) MotB-24DE (3.2°) mutants. The ∆*motB* strain does not assemble stators; the *motB*-24DE strain assembles stators and is motile (reproduced from [119] with permission). (**B**) FliF-FliG deletions alter C ring size. Size differences between *S. enterica* (**i**) CCW and (**ii**) CW-locked (FliG_∆__PAA_) C rings. (**iii.a**) The CW-biased *S. enterica* ∆FliF–FliG fusion assembles smaller C rings. (**iii.b**) 2D class averages from the ∆FliF–FliG fusion basal body; without and with the FliG_M_ D124Y residue substitution that restores normal bias (reproduced from [122] with permission).

The interactions of the FliGc domain with the MotA.MotB stator complexes determine torque. The biochemical studies of rotor–stator interactions in *E. coli* [11] were soon extended to other bacterial motors, notably the sodium-driven *V. alginolyticus* motor [114]. The FliG_C_ torque helix and adjacent segments in the *V. alginolyticus* motor contain more charged residues compared to *E. coli* [115]. Furthermore, substitutions at two residue positions selectively impair only CCW rotation [116]. Interestingly, the *V. alginolyticus* FliG_C_ also determines the correct polar localization and assembly of the stator complexes [117]. The mutagenesis of the residues involved in rotor–stator interactions in the *E. coli* motor was extended in the related *S. enterica* [118]. Notably, these investigators coupled fluorescent localization of the GFP-MotB fusion proteins with motility assays to parse out charged residues at the FliG_c_–MotA interface that influence stator assembly from those dedicated to torque generation. The first cryo-ET images documenting the alteration of C ring morphology by stator complexes have now been obtained in the spirochete *Borrelia burgdorferi*. Comparative study of *motB* mutant strains with defective versus restored proton conduction suggests that functional stators are required for full expression of this effect [119] (Figure 6A). Thus, torque affects switch architecture, in addition to CW/CCW rotation bias (Section 2), while the FliG rotor ting, in turn, affects assembly of the force-generating stator complexes.

The CCW and CW torque generated by stator interactions with the FliG ring must be transmitted via the intervening FliF MS-ring to the external components of the filament whose rotation is the physiologically relevant parameter. The *fliF* and *fliG* genes are adjacent in one operon for middle gene expression in the *S. enterica* flagellar regulon [120]. Strains with these fusions are motile and form C rings but have aberrant CW/CCW bias [121]. Their analysis has provided valuable clues on the effects of the FliF–FliG association on switch operation and C ring morphology. 

The bias of the full-frame fusion was restored by suppressor mutations in FliM_M_ and FliG_N_, as well as by insertion of a flexible, nine-residue glycine-rich linker at the fusion site [121], emphasizing the influence of FliG_N_ and FliG_M_ conformational plasticity on the sign of the transmitted torque. The deletion the FliF–FliG fusion (∆FliF.FliG) strain lacked a major part of FliG_N_,and formed smaller C rings with lower (n = 31) subunit stoichiometry [122]. In contrast, the FliG_M_∆PAA deletion strain formed smaller C rings with altered packing as their subunit stoichiometry was unchanged. The bias of the ∆FliF.FliG strain could be also be compensated by residue substitutions localized to the FliM_M_.FliG_M_ interface (FliG_D124Y_) and a likely FliM_M_ dimerization interface (F188Y, V186A). Cryo-electron microscopy of isolated FliG_D124Y_ basal bodies showed that D124Y substitution partly restored the C ring diameter towards wild-type values. These results demonstrate that either subunit number or spacing variation can change C ring size. While FliF.FliG_N_ association primarily transmits torque, it also has downstream effects on the switch machinery for torque reversal. There seems to be a synergistic relationship between the supramolecular organization of the FliG_N_ and FliM_M_ rings with defects in one restored by compensating alterations in the other (Figure 6B).

How does FliG_N_ transmit torque generated at the FliG ring periphery to the axial rod and filament via FliF? The architecture of the *S. enterica* hook basal body revealed by the work of DeRosier and colleagues (see [50] and references therein) notably shows different symmetries for the internal and external modules; the cytoplasmic C ring (n = 33–36) and the external hook (n = 11), for example. The architectural design by which torque transmission is achieved and symmetry mismatch accommodated is starting to be understood. The structural adaptation for torque transmission was determined in *T. maritima* [123] and, subsequently, the sodium-powered *V. alginolyticus* motor [124]. NMR reported extensive conformational changes in the *T. maritima* FliG_N_ ARM fold upon interaction with 46 FliF C-terminal residues. These changes were due to formation of the co-folded FliG_N_ and FiF C-tail domain. FliG_N_ in the crystallized co-folded domain alters conformation to closely match the FliG_M_ fold [125], and possibly also conformational plasticity compatible with the compensatory interactions between the two domains in the regulation of C ring size. The crystal structure of the *H. pylori* co-folded domain corroborated the essential features reported for *T. maritima* [126].

**Figure 7:**
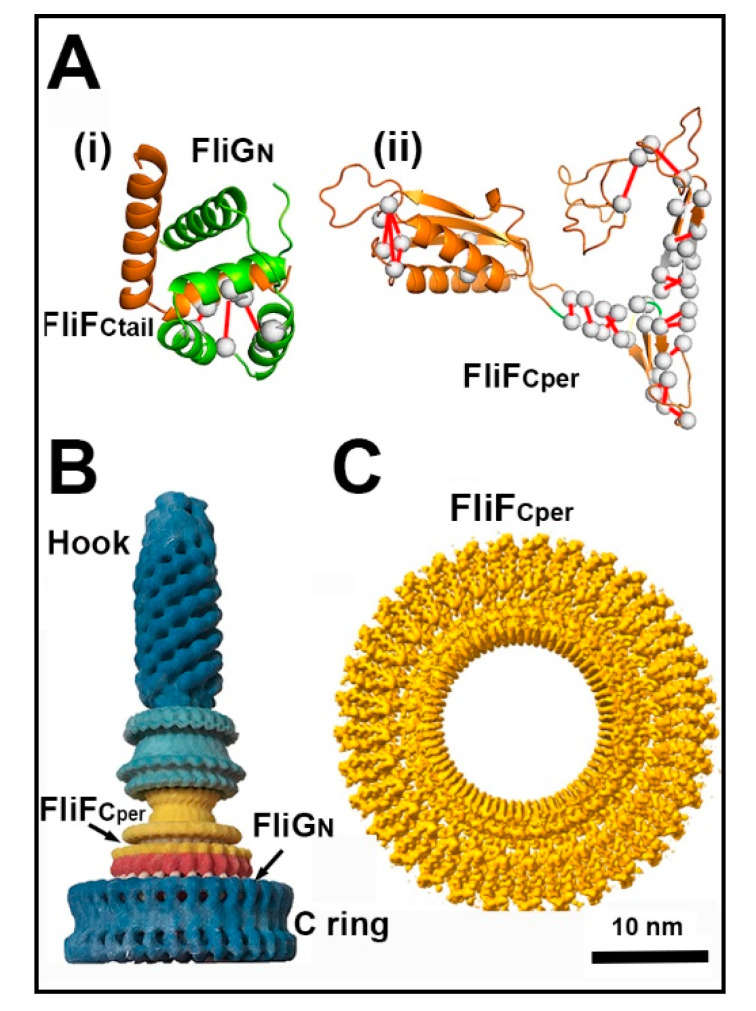
Transmembrane torque transmission. (**A**) (**i**) Conserved FliG_N_/FliF_C-tail_ coevolved contacts mapped onto the *T. maritima* crystal structure (PDB: 5tdy [125]). (**ii**) Predicted FliF_C_^per^ architecture obtained by residue coevolution. Red lines denote coevolved residue pairs as in Figure 5D (modified from [112]). (**B**) The 3D model of the *S. enterica* hook–basal body complex. The basal body part of the structure was from a 3D reconstruction as published [50]. The hook with attached FliD cap structure was done by single-particle methods using the entire hook with the cap as the single-particle (Dennis Thomas, unpublished results (with permission)). (**C**) The 3D map of the *S. enterica* FliF_C_ torque transmitter module (EMD-10143, 3.1-angstrom resolution (modified from [127])). The *en-face* view resolves 33-subunits.

The cytoplasmic FliF segment contiguous with the co-folded FliG_N_.FliF_C-tail_ domain is predicted to form a predominantly α-helical connector to the periplasmic C-terminal FliF (FliF_C_^per^) [112]. The recent 3D-cryoelectron microscopy reconstruction of overproduced *S. enterica* FliF rings has shown that FliF_C_^per^ forms a periplasmic scaffold with a split ring-building motif (RBM) that staples together an anti-parallel β-barrel to form the external periplasmic modules of the MS ring while the N-terminal FliF forms inner RBM modules structurally homologous to RBMs characterized for Type-III injectosomes [127]. Both the split RBM and the β-barrel were predicted by residue coevolution in the course of ongoing work on the full-frame FliF.FliG fusion ring [112]. Thus, the FliF flagellar motor scaffold has evolved to add a particularly stable, periplasmic C-terminal domain to the injectosome RBMs. The map further reveals that FliF_C_^per^ symmetry (n = 33–34), within the design tolerance reported for other biomolecular assemblies, matches the C ring symmetry to dispel the MS and C ring symmetry mismatch conundrum raised by initial estimates of a lower FliF subunit stoichiometry. Figure 7 summarizes these advances.

In summary, rotor–stator interactions employ a core set of charged residues and control large scale changes in stator assembly and C ring morphology either side of the interaction interface. The FliF_C_^per^ module presumably templates the assembly of the FliG ring via FliF_C-tail_ that is part of the co-folded domain. FliG_M_ then dictates FliM assembly via contacts with FliM_M_. The FliM_M_ ring drives reorientation of FliG_C_ for bidirectional torque generation and remodels in response to lesions in the co-folded FliG_N_.FliF_C-tail_ domain, Thus, there is conformational coupling of FliM_M_ to these distant domains via FliG_M_. The correlation between C ring size and rotation state, driven by defined lesions, is comparable to the rotation–fluorescence intensity correlation reported for GFP-tagged motors. It provides the first structural clue for the difference reported for the CW versus CCW torque–velocity relations.

## 4. Current Challenges

**Figure 8:**
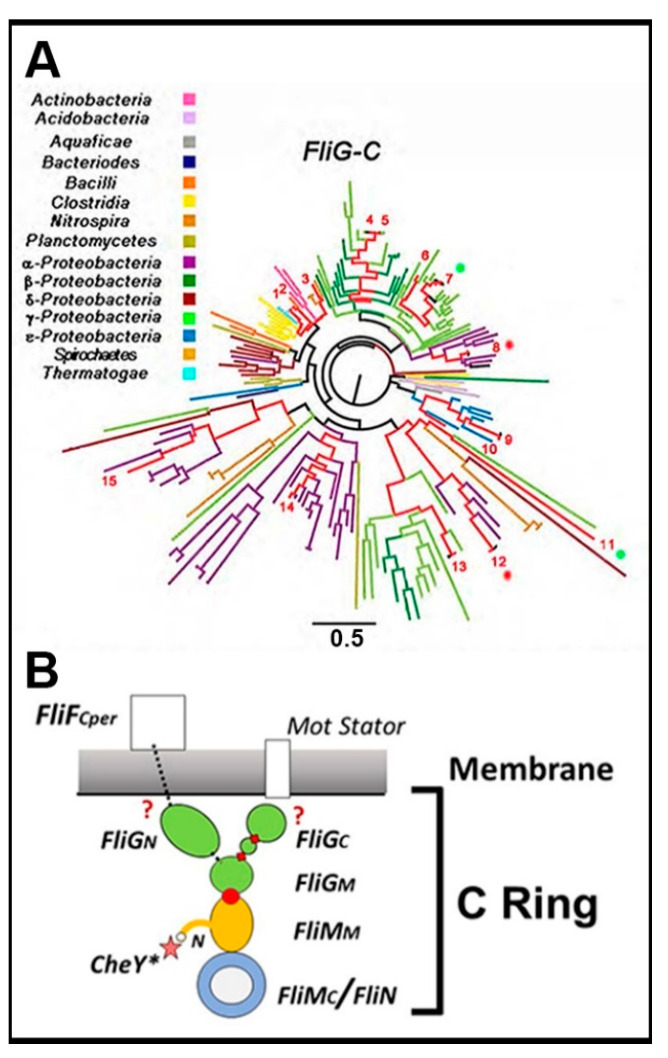
(**A**) The phylogenetic tree of the FliG_C_ motor domain. 160 seed sequences (multiple sequences from 23 species). Species (red lines), with well-studied flagellar biochemistry, physiology or structure (1 = *Thermatoga maritima*, 2 = *Bacillus subtilis*, 3 = *Borrelia burgdorferi*, 4 = *Escherichia coli,* 5 = *Salmonella enterica,* 6 = *Vibrio cholerae*, 7 = *Vibrio alginolyticus1*, 8 = *Rhodobacter sphaeroides1*, 9 = *Helicobacter pylori*, 10 = *Aquifex aeolicus*, 11 = *Vibrio alginolyticus2*, 12 = *Rhodobacter sphaeroides2*, 13 = *Vibrio parahaemolyticus*, 14 = *Caulobacter crescentus*, 15 = *Rhizobium meliloti*). Asterisks (*R. sphaeroides* (red), *V. alginolyticus* (green)) mark duplicates. (from ([110])). (**B**) The signal pathways in the BFM switch for the response to chemotactic stimuli, motor torque and load. The N-terminal FliM peptide (N) anchors activated CheY (CheY*) by a flexible tether and primes it to bind additional sites on the basal C ring (FliM/FliN). The bound CheY* modulates thermal FliM_N_ fluctuations for radial circumferential conformation spread and amplifies them via the conserved FliM_M_.FliG_M_ interface (red circle), and two hinges (red diamonds) bordering FliG ARM-C to reorient the peripheral FliGc Ca1-6 sub-domain. Inter-subunit stacking between the dynamic FliG_M_ and ARM-C modules stabilizes the FliG ring and regulates rotation reversal. The Mot stator complexes step along Ca1-6 to generate torque and influence FliG_MC_ dynamics. The conserved co-folded FliG_N_/FliF_C-tail_ domain ensures matching MS and C ring subunit stoichiometry. It is contiguous with the FliF C-terminal half (FliF_Cper_) that forms a stable assembly scaffold. Co-folded domain lesions/fusions have long-range effects on C ring architecture. Thus, FliG_M_ orchestrates bidirectional conformational coupling between FliM_M_ and either FliG_C_ or FliG_N_. The atomic details of the rotor–stator interactions or the linkage between FliG_M_ and FliF_Cper_ are poorly understood.

This review draws overwhelmingly from the *E. coli*, *S. enterica* and sodium *V. alginolyticus* motors for the elucidation of their molecular mechanisms, with a notable contribution from thermophile and *H. pylori* crystal structures. The fundamental issues regarding switch synchrony and ultra-sensitivity had been posed by 2003. The present knowledge of the structural basis for torque generation and transmission does not answer these issues but takes their study to a new level. Other species with diverse phylogeny have revealed the diversity of the switch operation. The bacillus *B. subtilis* has inverted motile responses to CheY. *V. alginolyticus* is one of several bacteria that alter switch frequency rather than rotation bias in response to CheY activation. The flagellum of the α-proteobacterium *R. sphaeroides* stops and starts. The thermophile *A. aeolicus* has been reported to lack FliM, consistent with its mostly smooth-swim motile behavior [128]. A major new challenge as illustrated by the phylogenetic tree of FliG_C_, the “motor” domain (Figure 8A), is to explain how such diversity can arise from a small core of protein components. 

Cryo-electron tomography (cryo-ET) has been an important role for the appreciation of the diverse morphology of flagellar motors (reviewed in [129,130]), even though it is presently limited to thin bacteria or mini-cells. Crucially, the technique provides 3D-reconstructions of complete motors that capture rotor–stator interactions [131], the effect of the cell membrane upon rotor flexure [132] and novel cell wall motor components [133]. Mutagenesis guided Cryo-ET has, interestingly, established diversification of C ring architecture for various cellular functions within a single species (*Campylobacter jejuni*) [133], and has led to the realization that high-torque motors have larger diameter stator and C rings [134]. 

The emergence of informatics due to developments in high-throughput sequencing, mass spectroscopy and high-performance computing is the highlight of this era, as appreciated by the increase in protein sequences (135,850 (2003) -> 177,754,527 (2020) (www.ebi.ac.uk/uniprot)) and atomic structures (<20,000 (2003) -> >140,000 (2018) [135]). Computational strategies for protein dynamic simulations [87,136] have benefited from the expanded databases. Most core switch components belong to a larger superfamily. The remarkable diversity of the CheY response regulator superfamily has been reviewed recently [137]. Several response regulators are expressed concurrently within one species. Many species have multiple CheYs of which typically one interacts with the flagellar motor: a study on *V. cholerae* being an early example [138]. FliF, FliM, FliN and FliY are members of larger families that include Type III injectosome components (reviewed in [139]). FliG has distant homology to the MgtE transporter family [140]. The diversity of the basal C ring and the CheY atomic structures suggests that bacteria utilize multiple strategies for signal reception.

The *E. coli* and *S. enterica* bacteria remain the primary source for motor rotation assays in conjunction with native and chimeric *V. alginolyticus* sodium motors. Structural knowledge, while anchored in these bacteria, now has important contributions from other species driven by the developments in cryo-ET and informatics. The current understanding of the flagellar motor switch is based on a generic, integrated assembly with contributions from multiple species, as schematized in Figure 8B.

Charged residues on a single FliG_C_ α-helix (torque helix) are the primary determinant of rotor–stator interactions. FliG_N_ co-folds with a FliF C-terminal fragment (FliG_N_.FliF_C-tail_) to form a domain with a similar architecture to FliG_M_; FliG_N_ full-frame or deletion fusions can be compensated by engineered modifications in the FliG_M_ domain. The co-folded domain connects to FliF_C_^per^, the assembly scaffold for the C ring. The matching 33–34 symmetries of FliF_C_^per^ and the C ring support the 1:1 stoichiometry for FliG_N_,FliF_C-tail_ seen in the crystal structures. A chained FliG_C_–FliG_M_ stack is the working model for rotor ring organization, but its assembly is probably modulated by other C ring components whose genes encode *mot* alleles [8,9]. There is not a straightforward match of the FliG subunit symmetry with the 26 steps per revolution resolved in rotation assays. Other stator–rotor contacts may determine step periodicity. The in-situ conformation and dynamics of the linkage between the chained FliG_C_–FliG_M_, the co-folded FliG_N_.FliF_C-tail_ domain and FliF_C_^per^ remain to be determined. 

The connectivity of the molecular linkage between the FliM_N_ CheY binding site and the FliG_C_ torque helix is now understood in broad outlines. FliM_N_ tethered the CheY associates with secondary binding sites on the basal C ring that may differ with species. FliM_M_ monomers fluctuate between bi-stable conformations that likely reflect the CCW and CW operational states. These states propagate across the C ring via inter-domain contacts and across a conserved interface to FliG_M_. FliG_M_ and its stacking contact with FliG_C_ ARM-C are dynamic. Helix_MC_ melting within the flexible inter-domain GG linker in conjunction with adjacent α-helices orchestrates these dynamics. The FliGc intra-domain MFXF hinge amplifies FliG_M_ motions to cause a large re-orientation of the FliG_C_ αC_3-6_ torque helix subdomain. These reorientations may reflect CCW <-> CW transitions. CW and CCW-locked motors have different torque velocity relations. Substitutions that alter the fold of FliG_C_ αC_3-6_ affect MFXF hinge dynamics and selectively impair CCW rotor-stator interactions. Residue substitutions that enhance CW or CCW bias have different FliG_M_ dynamics. Rotation state is influenced by changes in C ring size due to subunit number or packing variations. C ring morphology is also altered by the presence and activity of the stator complexes. The molecular basis of the coupling between FliG_M_ dynamics and CCW <–> CW transitions is not defined, let alone the coupling between the C ring dynamics and the torque–velocity relation. Adaptive subunit exchange of GFP-tagged basal-ring components may influence switch ultra-sensitivity to CheY activity. The C-ring is disrupted by single residue perturbations [141] and might also be impaired by GFP, which has a similar size to the proteins tagged. However, the CCW adaptive increase in basal body fluorescence is difficult to explain by GFP perturbation of C ring assembly. Nevertheless, the correlation between adaptation and subunit turnover is qualitative and determination of whether it is determinative or incidental must await structural elucidation of the turnover mechanism. Finally, the integrated picture is based on studies of a few species. The complete diversity of the species under current study is substantially more, as glimpsed in motor output, CheY function, basal C ring composition and C ring architecture. The determination of the conformational transitions of the stator complexes during the work cycle is a likely prerequisite for the explanation of their long-range effects on C ring morphology. More generally, elucidation, as opposed to description, of switch diversity is a severe challenge that may be intractable is the absence of the fundamental signal and energy transduction mechanism. 

Mathematical models have closely tracked the progress in motor physiology. The conformational spread model has been modified and extended, while a fundamentally different non-equilibrium motor model has been developed. These advances have led to the appreciation that switch operation cannot be understood in isolation from motor mechanics. However, the details of the chemical machinery that these models seek to explain has remained at the 2003 level. There is an urgent need for a top–down development of these models to discriminate between possible molecular mechanisms. MD simulations based on X-ray structures, NMR, ESR spectra and foot-printing techniques offer a bottoms-up approach to supply the kinetics required to correlate the molecular level descriptors known thus far with motor physiology. An atomic-level model of a rotor module from even one species will be a game-changer for a kinetic model with the necessary predictive power, although the scale-up of the simulations will be a logistic challenge with present-day resources. Nevertheless, research on this remarkable biomolecular machine has consistently advanced in unforeseen ways with fundamental implications for protein energetics and allostery. The innovation of its research community will ensure that it continues to do so.
